# Optogenetic inhibition of cocaine seeking in rats

**DOI:** 10.1111/j.1369-1600.2012.00479.x

**Published:** 2012-07-24

**Authors:** Michael T Stefanik, Khaled Moussawi, Yonatan M Kupchik, Kyle C Smith, Rachel L Miller, Mary L Huff, Karl Deisseroth, Peter W Kalivas, Ryan T LaLumiere

**Affiliations:** 1 Department of Neurosciences, Medical University of South CarolinaCharleston, SC, USA; 2 Departments of Bioengineering, Neurosurgery and Psychiatry, Howard Hughes Medical Institute and CNC Program, Stanford UniversityStanford, CA, USA; 3 Department of Psychology, University of IowaIowa City, IA, USA

**Keywords:** ArchT, eNpHR3.0, halorhodopsin, prelimbic cortex, nucleus accumbens core, reinstatement

## Abstract

Inhibitory optogenetics was used to examine the roles of the prelimbic cortex (PL), the nucleus accumbens core (NAcore) and the PL projections to the NAcore in the reinstatement of cocaine seeking. Rats were microinjected into the PL or NAcore with an adeno-associated virus containing halorhodopsin or archaerhodopsin. After 12 days of cocaine self-administration, followed by extinction training, animals underwent reinstatement testing along with the presence/absence of optically induced inhibition via laser light. Bilateral optical inhibition of the PL, NAcore or the PL fibers in the NAcore inhibited the reinstatement of cocaine seeking.

## INTRODUCTION

The present study applied inhibitory optogenetics to the circuitry underlying relapse to cocaine seeking and tested hypotheses regarding obligatory involvement of the projection from the prelimbic cortex (PL) to the core subcompartment of the nucleus accumbens core (NAcore) (Kalivas, Volkow & Seamans 2005). Pharmacological inactivation of either the PL or the NAcore prevents reinstated cocaine seeking (Kalivas *et al*. 2005). While this work implies a relationship between the PL and NAcore, pharmacological inactivation cannot isolate a role for axonal projections between nuclei.

Optogenetics allows functional analysis of connections between structures by optically controlling axon terminal fields rather than pharmacologically or electrophysiologically regulating nuclear cell groups to infer connections (Stuber *et al*. 2011). We employed *in vivo* optogenetic inhibition with either the chloride pump halorhodopsin (eNpHR3.0) or the outward proton pump archaerhodopsin (ArchT) to examine the PL-to-NAcore circuit in reinstated cocaine seeking. Both opsins have been previously shown to inhibit neural activity (Chow *et al*. 2010; Gradinaru *et al*. 2010), and, therefore, we explored the use of each in the inhibition of cocaine seeking. It was hypothesized that optogenetic inhibition of the PL, NAcore and the projection between the two nuclei inhibits reinstated cocaine seeking.

## MATERIALS AND METHODS (SEE APPENDIX S1 FOR DETAILS)

All methods used were in compliance with National Institutes of Health guidelines for care of laboratory animals and were approved by the Medical University of South Carolina or the University of Iowa Internal Animal Care and Use Committee. Male Sprague-Dawley rats were single, housed with a 12-hour reverse light/dark cycle. Animals were anesthetized and virus (rAAV2-hSyn-eNpHR3.0-EYFP, rAAV5-hSyn-EYFP, rAAV2-CAG-ArchT-GFP or rAAV5-CMV-GFP in |10^12^ viral molecules in 0.7 μl, University of North Carolina Vector Core) was stereotaxically delivered into the PL or NAcore. Twelve daily self-administration sessions lasted 2 hours or until the rats had taken a maximum of 200 infusions. Cocaine was self-administered using a Fixed Ratio 1 schedule with a 20-second timeout. Active lever presses produced a 0.05-ml infusion of 200 μg of cocaine and a 5-second tone/light cue. Rats underwent 10 d of extinction and then underwent either a cocaine- (10 mg/kg, ip) or a cocaine + cue-induced reinstatement where lever presses had no consequences. Rats underwent two reinstatement sessions for one or both types of reinstatement, counterbalanced with respect to whether illumination was provided. Illumination (10 mW of 561 nm light) was provided for the entire duration of the 2-hour reinstatement session. After reinstatement testing, animals underwent open-field testing followed by a cocaine injection (15 mg/kg, ip). Fiber optics were inserted, and either sham or laser illumination provided during the measurement of locomotor behavior (Deisseroth 2012).

Whole cell patch recordings were made in the PL or NAcore in rats previously microinjected with virus into each area. Pyramidal neurons or medium spiny neurons, respectively, were recorded (Moran *et al*. 2005). Extracellular field potentials were recorded in urethane anesthetized rats (Moussawi *et al*. 2009). Rats’ PL was microinjected with rAAV2-hSyn-eNpHR3.0-EYFP, concentric bipolar stimulating electrodes were placed in the PL, and glass recording electrodes were aimed at the NAcore.

Rats were transcardially perfused with 4% paraformaldehyde. Coronal sections were evaluated for yellow fluorescent protein (YFP) either by immunocytochemistry or using a helium/neon 543 nm laser line with a YFP excitation filter. Because a Kolmogorov–Smirnov test revealed that some of the reinstatement data were not normally distributed, a Friedman repeated measures nonparametric test was employed followed by a Dunn’s multiple comparison. The locomotor data were evaluated using a two-way analysis of variance with repeated measures over time, followed by a Bonferroni multiple comparisons test.

## RESULTS

### Validation of opsin location and function

**Figure 1:**
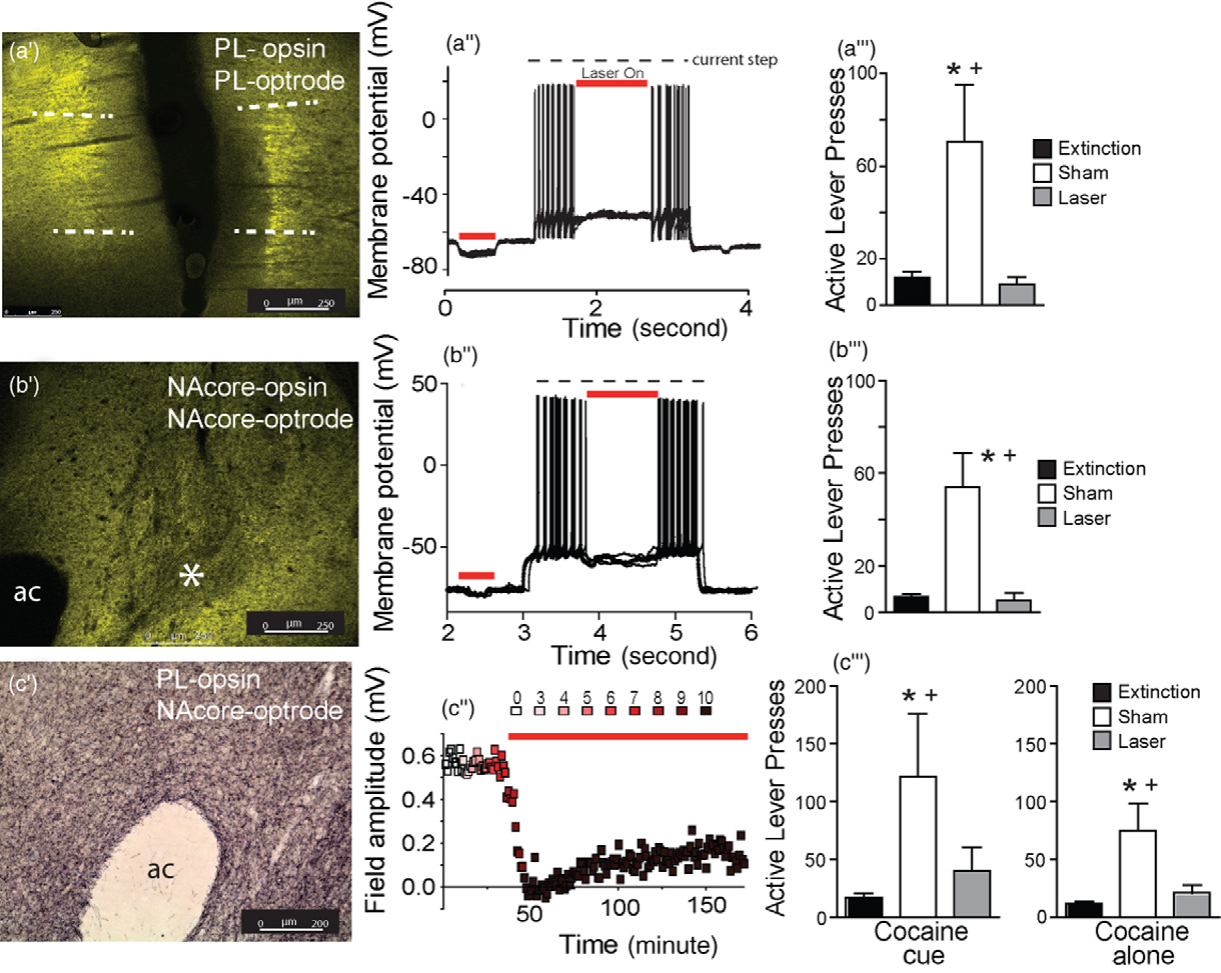
Histological and electrophysiological verification of the opsin expression and inhibition of reinstated cocaine seeking. (a’) eNpHR3.0-YFP expression (green) after virus injection into the prelimbic cortex (PL). Dashed lines indicate approximate boundaries of the PL. (a’’) Laser-mediated hyperpolarization and inhibition of firing in a single eNpHR3.0-infected pyramidal cell. (a’’’) Laser light in the PL suppressed cocaine + cue-induced reinstated behavior. (b’) eNpHR3.0-YFP expression in the nucleus accumbens core (NAcore) after virus injection into the NAcore. ac, anterior commissure. *Area of relatively low fluorescence corresponding to the location of the fiber optic. (b’’) Laser-mediated hyperpolarization and inhibition of firing of a single eNpHR3.0-infected medium spiny cell. (b’’’) Laser light into the NAcore suppressed reinstated behavior. (c’) Immunostaining of archaerhodopsin expression in axonal fibers the NAcore after virus injection into the PL. (c’’) Optical inhibition of field potentials recorded in NAcore after electrical stimulation in the eNpHR3.0-infected PL of a rat. Units of laser intensity are arbitrary and correspond to step increases until the field potential disappeared and remained at this intensity for the duration of the experiment. (c’’’) Laser illumination in the NAcore suppressed both cocaine- and cocaine + cue-induced reinstatement.

Figure 1 shows representative anatomical evidence for eNpHR3.0 expression in the PL (panel A’), eNpHR3.0 expression in NAcore neurons (panel B’) and ArchT expression in PL fibers terminating in the NAcore (panel C’). Whole cell patch clamp of a pyramidal neuron in the PL shows that optically inhibiting the infected region hyperpolarizes neurons and decreases action potential generation (Fig. 1a’’). Similarly, optically inhibiting NAcore medium spiny neurons reduced membrane potential and inhibited neuron firing (Fig. 1b’’). Figure 1c shows the results of optically inhibiting field potential amplitude in the NAcore that was elicited by electrically stimulating the PL. eNpHR3.0 was administered in the PL, and an optical fiber and recording electrode were inserted into the NAcore. Increasing laser intensity produced a progressive reduction in field amplitude. The capacity of activating eNpHR3.0 to reduce field potentials endured for at least 2 hours.

### Optical inhibition of PL and NAcore reduces reinstated cocaine seeking

Figure 1a’’’ shows that optical inhibition of eNpHR3.0-expressing PL reduced responding during cocaine-primed reinstatement (Friedman statistic = 11.19, *P* < 0.001, *n* = 7). Similarly, inhibition of the NAcore after intra-NAcore administration of either ArchT (*n* = 6) or eNpHR3.0 (*n* = 6) diminished responding during cocaine + cue-primed reinstatement sessions (Fig. 1b’’’). A Kruskal–Wallis test revealed no significant difference in responding between the ArchT or eNpHR3.0 animals, and thus, the data from these groups were pooled for analysis (Friedman statistic = 18.17, *P* < 0.001, *n* = 12). Figure 1c’’’ shows that optical inhibition of ArchT-expressing PL fibers in the NAcore reduced cocaine seeking induced by either a cocaine-priming injection (10 mg/kg; Friedman statistic = 9.80, *P* = 0.006, *n* = 10) or a combination cocaine-priming + cues (Friedman statistic = 9.90, *P* = 0.003, *n* = 10). In no experiment was inactive lever pressing affected by laser stimulation of fibers in the NAcore (data not shown).

#### Control virus experiments

Figure S1 shows that laser stimulation of cells in the NAcore infected with control virus or fibers in the NAcore following PL infection had no effect on cocaine alone or cocaine + cue-reinstated cocaine seeking, indicating that heating from the continuous laser light did not affect cocaine-seeking behavior. Figure S2 shows that optical inhibition of the NAcore, as in Fig. 1B, did not affect open-field behavior or cocaine-induced locomotor activity, suggesting the lack of these manipulations on nonspecific motor behavior. Inactive lever presses were not affected by laser stimulation in either the PL or NAcore groups (data not shown).

### Histology

Figure S3 shows the location of optical fiber placement in each experiment.

## Discussion

The present study is the first to use optogenetic inhibition to examine the neural circuitry underlying reinstated drug seeking and demonstrates that optogenetically inhibiting neurons in the PL or NAcore, or PL axons terminating in the NAcore prevents the reinstatement of cocaine seeking. This finding validates previous claims using pharmacological inhibition of the NAcore and PL that this projection is critical to reinstated cocaine seeking (see Introduction) and demonstrates the utility of optogenetic inhibition for characterizing the neural circuitry underlying cocaine seeking in greater detail than was previously possible using pharmacological inhibition or electrical stimulation.

## Supporting Information

**Figure S1** Control virus-infected rats show no effect in response to laser stimulation **Figure S2** Locomotor activity in a novel open-field or following a cocaine injection (15 mg/kg, ip) was not altered by optical activation of archaerhodopsin (ArchT) in the nucleus accumbens core (NAcore) of animals previously microinjected with adeno-associated virus-ArchT in the NAcore. **Figure S3** Location of optical fibers in the prelimbic cortex (PL) or nucleus accumbens core (NAcore). **Appendix S1** Supplemental methods.
