# Supplementary material for: Optogenetic inhibition of cocaine seeking in rats
Source: Addict Biol. 2012 Jul 24;18(1):50–3. doi: 10.1111/j.1369-1600.2012.00479.x (PMC3578202; doi:10.1111/j.1369-1600.2012.00479.x)
Supplement: Supplementary file 1 — Figure S1 Control virus-infected rats show no effect in response to laser stimulation Figure S2 Locomotor activity in a novel open-field or following a cocaine injection (15 mg/kg, ip) was not altered by optical activation of archaerhodopsin (ArchT) in the nucleus accumbens core (NAcore) of animals previously microinjected with adeno-associated virus-ArchT in the NAcore. Figure S3 Location of optical fibers in the prelimbic cortex (PL) or nucleus accumbens core (NAcore). Appendix S1 Supplemental methods. [file adb0018-0050-sd1.docx]

**SUPPLEMENTAL METHODS**

**Animal housing and surgery**

All methods used were in compliance with NIH guidelines for care of laboratory animals and were approved by the Medical University of South Carolina Institutional Animal Care and Use Committee or the University of Iowa Institutional Animal Care and Use Committee. Male Sprague-Dawley rats (250-300g) (Charles River Laboratories, Indianapolis, IN) were single housed under controlled temperature and humidity with a 12 hour reverse light/dark cycle (lights on at 18:00). Rats were fed ad libitum until 7 days after surgery, after which food was restricted to 4 chow pellets/day. Water was always available ad libitum.

Rats underwent surgeries for injection of virus, implantation of cannulae, and implantation of catheters. For surgeries, animals were anesthetized with ketamine HCl (87.5 mg/kg Ketaset, Fort Dodge Animal Health) and xylazine (2mg/kg Rompum, Bayer). For microinjections of virus, 0.7µl virus (rAAV2-hSyn-eNpHR3.0-EYFP or rAAV5-hSyn-EYFP or rAAV2-CAG-ArchT-GFP or rAAV5-CMV-GFP ~10^12^ viral molecules/mL, UNC Vector Core) was delivered bilaterally (0.7µL/min for 1 min) through a 33 gauge needle. The needles were left in place for 8 minutes following injection to allow diffusion of viral particles away from the injection site and then slowly retracted. For viral injections into the PL or NAcore, the coordinates were as follows: PL (double-barreled microinjector), AP: +3.1 mm from Bregma, ML: ±0.6 from midline, and DV: -3.9 mm from skull surface; NAcore, AP: +1.5 mm and ML: ±3.0 mm from midline and DV: -5.2 mm from skull surface at a 9° angle based on Paxinos and Watson ([2005](#_ENREF_11)).

Catheters were implanted, as described previously ([LaLumiere et al., 2012](#_ENREF_7)). Catheters were flushed daily with cefazolin (0.2 mL of 0.1 gm/mL) and heparin (0.2mL of 100 IU) for 1 wk, then daily with heparin for the remainder of the experiment to prevent infection and maintain catheter patency. Rats were then stereotaxically implanted with bilateral guide cannulae (20 gauge; Plastics One) aimed at the PL or NAcore. Guide cannulae were lowered into place at an 18^o^ angle and attached to the skull via small screws and dental acrylic. Obdurators were used to prevent obstruction of the cannulae. Animals recovered for one week before behavioral training.

**Self-administration, extinction and reinstatement procedures**

All self-administration experiments occurred in standard operant chambers (Med Associates, Fairfield, VT) with two retractable levers, a house light, and a cue light and tone-generator (2900 Hz). Prior to drug self-administration, all rats were food-deprived for 24 h and then underwent a single 15-h food-training session in which the rats were trained to press the active lever for a single food pellet (45 mg, Noyes, Lancaster, NH) on a fixed-ratio 1 (FR1) schedule. Following the food training, rats were given ~20 g of food immediately after every self-administration session, and this food restriction was maintained through the experiment. One day after the food training, the rats began cocaine self-administration and each session lasted 2 h or until the rats had taken a maximum of 200 infusions. The self-administration program was an FR1 schedule with a 20 s timeout to prevent overdose. Each active lever press produced a 0.05 ml infusion of 200 µg of cocaine (dissolved in 0.9% sterile saline; cocaine-HCl kindly provided by NIDA). Concurrent with the drug infusion, a cue tone (2900 hz) and cue light immediately above the active lever turned on for 5 s. Rats underwent self-administration 6 d/week for at least 2 weeks (i.e. 12 days). In order to transition into extinction training, rats were required to take at least 10 infusions of cocaine over 10 days, including the last 3 days of self-administration, as well as discriminate between the active and inactive lever. If rats had not met these criteria by 4 weeks (i.e. 24 days), they were excluded from the study.

After self-administration, rats began extinction training. Active lever presses produced no drug infusion or light/tone cues. For all experiments, rats underwent at least 10 d of extinction. Because the optical manipulation required attaching a leash to cannulae on the rats’ head, rats underwent two extinction sessions with leashes attached prior to reinstatement testing in order to accustom the rats to the leash. These sessions served as the extinction baseline for comparison. Rats then underwent either a cocaine-prime reinstatement or a cocaine+cue reinstatement. In both cases, rats received a single injection of cocaine (10 mg/kg) immediately prior to the session. For the cocaine-induced reinstatement, lever presses had no consequences. For the cocaine+cue reinstatement, active lever presses produced the combination light/tone cues that had been present during self-administration. Rats did not receive intravenous drug infusions and underwent two reinstatement sessions for one or both types of reinstatement, counterbalanced with respect to whether illumination was provided.

**Locomotor activity**

After reinstatement testing, animals underwent open field testing followed by a cocaine injection (15 mg/kg, ip) to examine the effects of optical illumination on locomotor activity. Motor activity was monitored in clear plexiglass boxes and photobeam breaks were detected and counted by a computer running Digiscan software (AccuScan Instruments Inc., Columbus, OH). Bare optical fibers were inserted and either sham or laser illumination provided over the 60 min measurement period after the animal was placed into the open field or after administration of cocaine.

**Optical inhibition**

Optical probes were constructed in which the fiber optic (200 µm core, multimode, 0.37 NA) was inserted and glued into a 24 ga internal cannula. The fiber extended 0.5 mm beyond the end of the internal cannula. During the experiment, the optical probe was inserted into the guide cannula that had been previously implanted during surgery. The internal cannula extended 1 mm beyond the end of the guide cannula. Thus, the optical probe terminated ~0.5 mm prior to the site intended to receive light. The fiber optic probe was inserted through a leash that attached to the guide cannula, enabling a secure attachment to the rat that prevented the fiber from being chewed. The other end of the fiber optic (FC/PC connection) was attached to a fiber splitter (2x1) that permitted simultaneous, bilateral illumination. The single end of the splitter was attached to a rotating optical commutator (Doric Lenses) to permit free movement of the rat. The commutator connected to a fiber that connected to a laser (DPSS, 200 or 300mW, 561nm, with a multimode fiber coupler for an FC/PC connection, OEM Laser Systems). Prior to the experiments, the light output of the fiber optic was adjusted to approximately 10 mW of light, as measured by an optical power meter. Based on measurements made in mammalian brain ([Deisseroth, 2012](#_ENREF_4)), assuming a geometric loss of light, light output of 10 mW measured by a standard optical power at the tip of a fiber with an NA of 0.37 and a fiber core radius of 200 µm will produce ~ 1 mW/mm^2^ of light up to 1 mm directly away from the fiber tip, which is the minimum amount necessary to produce opsin activation ([Gradinaru et al., 2009](#_ENREF_5); [Tye et al., 2011](#_ENREF_13)), though evidence suggests that even lower levels of irradiance (e.g 0.35 mW mm^2^) are sufficient for ArchT activation ([Chow et al., 2010](#_ENREF_3)). Based on in vivo measurements of the shape of the light output in mammalian brain tissue, these parameters would be expected to provide sufficient light for opsin activation in at least 0.4 mm^3^ of tissue ([Yizhar et al., 2011](#_ENREF_14)), though the amount of brain tissue optically inhibited at least partially with ArchT could be higher ([Chow et al., 2010](#_ENREF_3)).

**Immunohistochemistry and confocal imaging**

Rats were anesthetized by pentobarbital (100mg/ml, ip) and then transcardially perfused with phosphate-buffered saline (PBS, pH 7.4) followed by PBS containing 4% (w/v) paraformaldehyde. Brains were post-fixed for 24 hours at room temperature in perfusion solution. Coronal sections (50µm thick) were incubated for 60 minutes in 1% hydrogen peroxide, rinsed 3 times in PBS, and then incubated overnight in PBS containing 0.25% triton-X, 0.01% sodium azide, and anti-GFP (rabbit, 1:50,000, Abcam) antibody. Sections were rinsed once in PBS and incubated in PBS containing the biotinylated secondary antibody (donkey, 1:1,000, Jackson Immunoresearch) for 30 minutes, rinsed 4 times in PBS, and incubated for 1 hour in an ABC Kit (Vector Labs). Sections were then rinsed once in PBS and incubated in PBS+0.05% diaminobenzidine with 0.05% hydrogen peroxide for 5 minutes. Slices were then mounted and ArchT expression was visualized on light microscope. In other experiments the YFP tag was visualized in brain sections using a Leica confocal microscope. The fluorescent tag was excited using a Helium/Neon 543nm laser line with a YFP or GFP excitation filter. Images were scanned at a system-optimized thickness along the z-axis, and the frame size for images was set at 1024x1024 pixels.

**In vivo and whole cell patch recordings**

Whole cell patch recordings (Figure 1A’’ and B’’) were made in the PL or NAcore in rats previously microinjected with virus into each area. Slices were made of the PL or NAcore and pyramidal neurons or medium spiny neurons, respectively, were recorded ([Moran et al., 2005](#_ENREF_9); [Shen et al., 2011](#_ENREF_12)). Slices were placed in the recording chamber and the fluorescent area in the NAcore or PL was identified using a fluorescent illumination system X-Cite 120 PC (EXFO, Ontario, Canada) with a Fluorescein-Isothiocyanate (FITC) filter (Olympus America, Center Valley, Pennsylvania). Neurons were visualized in the area of highest fluorescence with an Olympus BX51WI microscope. Action potentials were evoked by a current injection into the soma by the patching electrode. An optic fiber was positioned close to the slice, just above liquid level, and the laser (561 nm) was activated by the Axograph X software.

Extracellular field potentials (Figure 1C’’) were recorded in urethane (1.5 g/kg, i.p.)-anesthetized rats (Moussawi et al., 2009). Briefly, rats were microinjected into the PL with rAAV2-hSyn-eNpHR3.0-EYFP, and concentric bipolar stimulating electrodes (Rhodes medical Instruments) were placed in the PL (AP +3.0mm; ML +0.6mm; DV -3.3mm from brain surface). Glass recording electrodes were pulled (1-2 mega ohm), and filled with 0.5M sodium acetate with 2% pontamine sky blue. Recording electrodes were aimed at the dorsomedial region of the NAcore (AP +1.8mm; ML +1.3 to 1.5mm; DV -5.5 to -6.2mm from brain surface) (Paxinos and Watson, 2006), and targeted just ventral to the tip of a previously inserted optical fiber. As described in detail elsewhere ([Moussawi et al., 2009](#_ENREF_10)), extracellular field potentials were amplified and the data band-pass filtered at 300Hz, then digitized by a National Instruments PCM-C1016E4 board (Austin, Texas) and analyzed using Custom Labview Software (Lee Campbell, Salk Institute, La Jolla, CA). Data were collected every 30 sec, at a 10kHz sampling frequency, and then averaged every 1 minute. Pulse width was set to 0.3 msec and basal stimulation intensity corresponded to ~50% of minimum current intensity that evoked a maximum field response. Field potential amplitude was measured as the difference between the mean of a 2-4 msec window prior to the stimulation artifact and the mean of a 1msec window around 15 msec following the stimulation artifact. After collecting baseline field potentials, the laser was turned on and continuous illumination was provided. The intensity of the light was increased over 4 min intervals (arbitrary units color-coded in Figure 1C’’) until the field potential was abolished (~ 10mW light output). This intensity was maintained for the duration of the experiment (120 min).

**Data Analysis**

Since a Kolmogorov-Smirnov test revealed that some of the reinstatement data were not normally distributed, a Friedman repeated measures nonparametric test was employed followed by a Dunn’s multiple comparison. The locomotor data were evaluated using a two-way ANOVA with repeated measures over time, followed by a Bonferroni multiple comparisons test. Data were analyzed in Prism (GraphPad Software). Statistical significance level was p ≤ 0.05, and data are presented as mean ± SEM.

**SUPPLEMENTAL DATA**


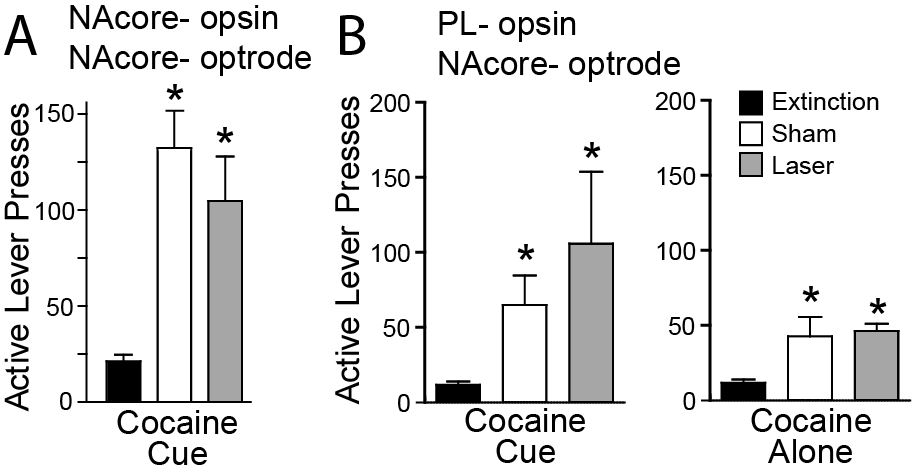


**Figure S1.** Control virus infected rats show no effect in response to laser stimulation. **A.** Laser light of the same intensity and duration as used in for active virus experiments did not alter cocaine+cue-induced reinstatement when animals were microinjected with control virus into the NAcore and optrode stimulation conducted in the NAcore. (Friedman statistic=11.14, p=0.001, n=7). The control data were pooled from two control virus, rAAV5-CMV-GFP (n=4, ArchT control) and rAAV5-hSyn-EYFP (n=5, eNpHR3.0 control). **B.** Illumination of control rAAV5-CMV-GFP infected fibers in the NAcore had no effect on either cocaine alone or cocaine+cue reinstated cocaine-seeking (Figure 3B; Friedman statistic=6.40, p=0.039, n=5).

*p< 0.05 compared to extinction levels of active lever pressing.

**
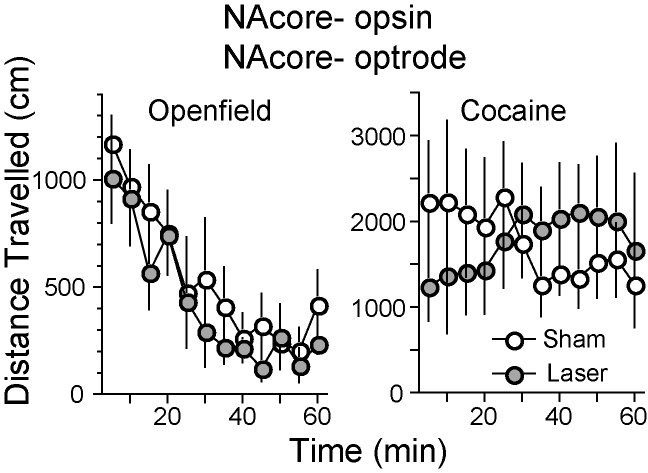
**

**Figure S2.** Locomotor activity in a novel open field or following a cocaine injection (15 mg/kg, ip) was not altered by optrode activation of ArchT in the NAcore of animals previously microinjected with AAV-ArchT in the NAcore. Because optical stimulation of NAcore neurons produced such a marked reduction in lever pressing, and the NAcore has a well-established role in processing and integrating motor behaviors ([Kelley, 2004](#_ENREF_6); [McFarland and Kalivas, 2003](#_ENREF_8)), we examined the capacity of optical inhibition to reduce locomotor activity. The same intensity of laser stimulation that abolished reinstated cocaine-seeking did not affect open field behavior (2-way ANOVA with repeated measures over time, time F[11,11]=9.67, p<0.001, no effect of treatment group or interaction between time and treatment; n=5 in each group) or cocaine-induced locomotor activity (no effect of time, treatment or interaction, n=5).

*p< 0.05 compared to extinction levels of active lever pressing.

+p< 0.05 comparing laser to sham treatments


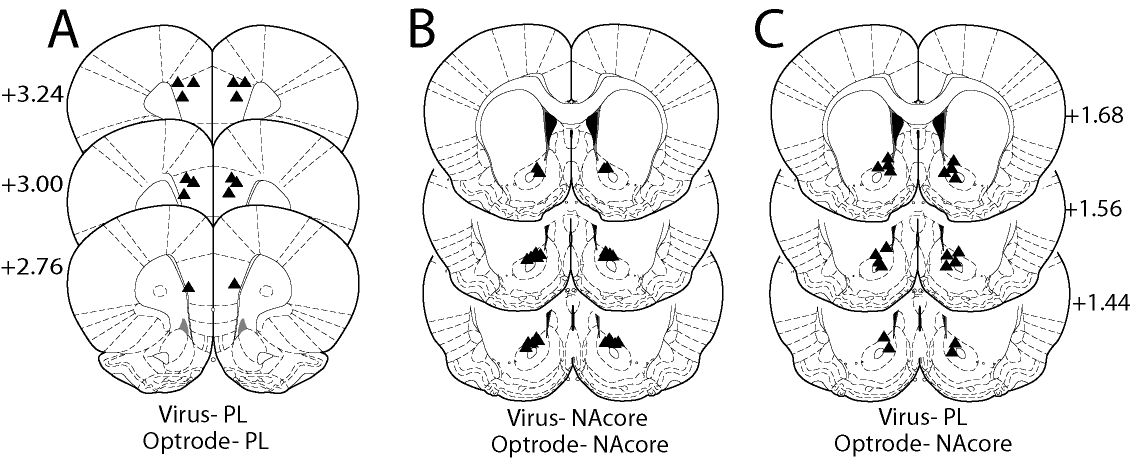


**Figure S3.** Location of optrodes in the PL or NAcore. The triangle shape approximates the predicted perimeter of the diffusion of laser light in the brain that expands in a cone shape for approximately 0.5 mm in length and 0.5 mm in diameter at the most ventral penetration ([Adamantidis et al., 2007](#_ENREF_1); [Aravanis et al., 2007](#_ENREF_2)). Accordingly, in figure 4 the apex of the triangle corresponds to the histologically identified ventral point of the optrode and the triangle illustrates the approximate shape and size of the illuminated tissue. **A.** Location of optrodes within the PL of animals microinjected with AAV into the PL from experiment shown in figure 1A. The predicted area of inhibition was largely in the dorsal half of the PL. **B.** Location of optrodes within the NAcore of rats microinjected with AAV into the NAcore from experiment shown in figure 1B. The predicted area of inhibition was largely in the dorsal half above the anterior commissure for the experiment when the virus was injected into the NAcore. **C.** Location of optrodes in the NAcore of rats microinjected with AAV into the PL from experiment shown in figure 1C. The predicted area of inhibition was largely in the dorsomedial quadrant when the virus was injected into the PL and axon terminals in the NAcore illuminated. Schematics of coronal sections were taken from Paxinos and Watson (2005). The numbers refer to location of the coronal section in mm from Bregma.

**REFERENCES**

Adamantidis AR, Zhang F, Aravanis AM, Deisseroth K, de Lecea L (2007) Neural substrates of awakening probed with optogenetic control of hypocretin neurons. Nature 450:420-424.

Aravanis AM, Wang LP, Zhang F, Meltzer LA, Mogri MZ, Schneider MB, Deisseroth K (2007) An optical neural interface: in vivo control of rodent motor cortex with integrated fiberoptic and optogenetic technology. J Neural Eng 4:S143-156.

Chow BY, Han X, Dobry AS, Qian X, Chuong AS, Li M, Henninger MA, Belfort GM, Lin Y, Monahan PE, Boyden ES (2010) High-performance genetically targetable optical neural silencing by light-driven proton pumps. Nature 463:98-102.

Deisseroth K (2012) Predicted irradiance values: Model based on direct measurements in mammalian brain tissue.

Gradinaru V, Mogri M, Thompson KR, Henderson JM, Deisseroth K (2009) Optical deconstruction of parkinsonian neural circuitry. Science 324:354-359.

Kelley AE (2004) Ventral striatal control of appetitive motivation: role in ingestive behavior and reward-related learning. Neurosci Biobehav Rev 27:765-776.

LaLumiere RT, Smith KC, Kalivas PW (2012) Neural circuit competition in cocaine-seeking: roles of the infralimbic cortex and nucleus accumbens shell. The European journal of neuroscience 35:614-622.

McFarland K, Kalivas PW (2003) Motivational systems. In: *Hanbook of Psychology*. Gallagher M, Nelson RJ (eds). John Wiley & Sons, Inc: Hoboken, NJ. pp 379-404.

Moran MM, McFarland K, Melendez RI, Kalivas PW, Seamans JK (2005) Cystine/glutamate exchange regulates metabotropic glutamate receptor presynaptic inhibition of excitatory transmission and vulnerability to cocaine seeking. J Neurosci 25:6389-6393.

Moussawi K, Pacchioni A, Moran M, Olive MF, Gass JT, Lavin A, Kalivas PW (2009) N-Acetylcysteine reverses cocaine-induced metaplasticity. Nat Neurosci 12:182-189.

Paxinos G, Watson C (2005) *The Rat Brain in Stereotaxic Coordinates*. 5th ed. Elsevier Academic Press: Amsterdam.

Shen H, Moussawi K, Zhou W, Toda S, Kalivas PW (2011) Heroin relapse requires long-term potentiation-like plasticity mediated by NMDA2b-containing receptors. Proceedings of the National Academy of Sciences of the United States of America 108:19407-19412.

Tye KM, Prakash R, Kim SY, Fenno LE, Grosenick L, Zarabi H, Thompson KR, Gradinaru V, Ramakrishnan C, Deisseroth K (2011) Amygdala circuitry mediating reversible and bidirectional control of anxiety. Nature 471:358-362.

Yizhar O, Fenno LE, Davidson TJ, Mogri M, Deisseroth K (2011) Optogenetics in neural systems. Neuron 71:9-34.
